# Technique of ultrasound-guided peripheral venous access in the emergency room

**DOI:** 10.1186/cc12200

**Published:** 2013-03-19

**Authors:** MG Annetta, G Scoppettuolo, M Biancone, F Toni, M Pittiruti

**Affiliations:** 1 Catholic University, Rome, Italy

## Introduction

In emergency situations, patients may need a fast and reliable peripheral venous access, which sometimes may be difficult to obtain, because of poor visualization of the superficial veins due to edema, obesity, hypovolemia or local abnormalities. In such cases, insertion of a central line is potentially time consuming and possibly associated with complications. Furthermore, central lines inserted in emergency are known to be at high risk of infection, so guidelines recommend that they should removed within 24 to 48 hours. In this setting, ultrasound-guided placement of a peripheral venous access might be more rapid, safer and more cost-effective than a central line.

## Methods

We have reviewed retrospectively our experience with the emergency use of 18 G or 20 G polyurethane catheters, 8 to 10 cm long, inserted by direct Seldinger technique.

## Results

In 1 year, 76 long peripheral catheters were inserted in emergency conditions, using ultrasound guidance. The success rate was 100%; most lines lasted >1 week and were used for different purposes, including contrast medium injection.

## Conclusion

The direct Seldinger technique allows a rapid and safe placement of the catheter in a vein of the arm or of the forearm, even when the vessel cannot be palpated or seen, as long as it can be visualized by ultrasound and it is not deeper than 2 cm. The long life of this type of peripheral line (up to 2 weeks) is guaranteed by the material (polyurethane being more biocompatible than Teflon) and by the length of the catheter (which reduces the risk of dislodgment). Also, these catheters are particularly cost-effective if compared with a central line or with a midline catheter, since a complete kit including catheter, 20 G needle and 20 cm guidewire costs between €15 and €20.

